# Lethal cardiotoxicity, steatohepatitis, chronic pancreatitis, and acute enteritis induced by capecitabine and oxaliplatin in a 36-year-old woman

**DOI:** 10.1186/1746-1596-8-150

**Published:** 2013-09-16

**Authors:** Simona Gurzu, Ioan Jung, Maria Comsulea, Zoltan Kadar, Leonard Azamfirei, Calin Molnar

**Affiliations:** 1 Department of Pathology, University of Medicine and Pharmacy of Tirgu-Mures, Tirgu-Mures, Romania; 2 Department of Oncology, County Hospital of Tirgu-Mures, Tirgu-Mures, Romania; 3 Intensive Care Unit, University of Medicine and Pharmacy of Tirgu-Mures, Tirgu-Mures, Romania; 4 Department of Surgery, University of Medicine and Pharmacy of Tirgu-Mures, Tirgu-Mures, Romania

**Keywords:** Capecitabine, Oxaliplatin, Cardiotoxicity, Chronic pancreatitis, Steatohepatitis, Enteritis, Multiorgan toxicity, Myocardial fibrosis, Lethal, Pericarditis

## Abstract

**Abstract:**

A 36-year-old female was hospitalized with symptoms suggesting intestinal occlusion. She was diagnosed with adenocarcinoma of the ampulla of Vater (pT4N0 stage) and underwent cephalic duodenopancreatectomy 8 months ago. Five cycles of postoperative chemotherapy were administrated using capecitabine and oxaliplatin (CAPOX or XELOX), the last one being completed 1 month ago. During the present hospitalization, because of normal computed tomography and ultrasound abdominal examination, rehydration and antibiotherapy were administrated. However, 4 days after hospital admission, the patient died. At autopsy and histological examination, we found a severe myocardial sclerosis with large scarring areas, severe steatohepatitis, chronic pancreatitis with large fibrotic areas, and acute enteritis. Alcohol consumption was denied. The patient died due to associated heart, liver and pancreatic failure. This multiorgan toxicity and death following CAPOX regimen had not yet been reported in the literature.

**Virtual slides:**

The virtual slide(s) for this article can be found here: http://www.diagnosticpathology.diagnomx.eu/vs/6472150549833105

## Introduction

In advanced gastrointestinal (GI) cancers, an association between cytotoxic drugs and biological agents is usually used. The biological agents such as bevacizumab, cetuximab, and panitumumab seem to be well tolerated and exhibit promising results, but their cost-effectiveness has been seriously evaluated in the literature. Bevacizumab, an antiangiogenic drug, was approved by the Food and Drug Administration (FDA) in 2004 for the treatment of colorectal carcinoma. Cetuximab and panitumumab, the anti-EGFR (epidermal growth factor) agents, can also be used in case of GI tumors that do not display K-ras mutation [1]. However, these agents cannot be used alone but only associated with classical chemotherapeutics, and have been approved only for metastatic cases, with the adjuvant protocol for locally advanced cancer being questionable [1].

In most of the GI locally advanced or metastatic tumors, cytotoxic drugs such as irinotecan, oxaliplatin, 5-fluorouracil (5-FU), and capecitabine are used as first-line therapy [1,2]. Oxaliplatin was approved by the FDA in 2002 for the treatment of Stage III/IV (Dukes’ C/D) colorectal carcinoma [2], but it is also used in other adenocarcinomas of the GI tract. Depending on the patient’s functional status and comorbidities, different combination regimens are used, such as FOLFIRI (5-FU, leucovorin [folinic acid], irinotecan), FOLFOX (5-FU, leucovorin [folinic acid], oxaliplatin), and GEMOX (gemcitabine, oxaliplatin); in addition, monotherapy treatments are also utilized (5-FU, gemcitabine, capecitabine) [1-3]. As some clinical trials revealed significant cardiotoxicity in case of intravenous 5-FU, when compared with oral fluoropyrimidine also known as capecitabine, and similar or superior efficacy in case of capecitabine, the oral administration of capecitabine is preferred in association with oxaliplatin (XELOX or CAPOX) [2-5].

In locally advanced cases, at least two problems should be taken into account. On the one hand, the predictive criteria are not well defined, with the microsatellite status or immunostaining being more valuable as prognostic than predictive factors [6]. On the other hand, despite the benefits of adjuvant therapy, its dose-dependent increased toxicity and costs should also be taken into account to select patients who are likely to benefit from them [1]. In this study, we present a multiorgan fatal chemotherapy toxicity, in a 36-year-old female suffering from locally advanced adenocarcinoma of the ampulla of Vater. In addition, with regard to the rarity of this lesion, a review of the relevant literature regarding chemoterapy-induced toxicity has been carried out.

## Case presentation

A 36-year-old cachectic female (body weight was 41.2 kg and height was 1.62 m), previously diagnosed with adenocarcinoma of the ampulla of Vater that invades the pancreas (pT4N0 stage), which was surgically removed (cephalic duodenopancreatectomy, radical surgery) 8 months ago, was admitted to our hospital with pale skin, scleral jaundice, sudden onset of severe abdominal pain and cramping, vomiting, diarrhea, and subfebrility. No alcohol, tobacco, or other drug use was declared. Before and after surgery and also before adjuvant therapy, no comorbidities were diagnosed, and the general status was carefully assessed without modification. Two months following surgery, she underwent five chemotherapy cycles with a combined regimen that included oral capecitabine and intravenous oxaliplatin (CAPOX or XELOX). CAPOX regimen comprised administration of 150 mg/m^2^ of oxaliplatin (diluted in a 5% glucose solution) intravenously over 120 min on Day 1, and then 1250 mg/m^2^ of oral capecitabine administered twice daily from Days 1 to 14, followed by 1-week drug holiday, in a 21-day cycle. The last (fifth) cycle was stopped 1 month before the present admission. The reason for choosing this aggressive regimen was the patients’ age and good pre-chemotherapy general status. There were no significant acute toxicity-related disorders, except slight diarrhea and vomiting, either of which recurred before this episode.

During the present admission, on physical examination, when palpating the abdomen, generalized abdominal tenderness with voluntary guarding was observed, with hypoactive bowel sounds. Considering her symptoms that suggested a tumor relapse, we decided to perform an emergency GI endoscopy that did not evidence tumor relapse or intestinal obstruction. The abdominal-computed tomography and ultrasound were normal. Her serology showed slight anemia (hemoglobin: 10 g/dl, hematocrit: 40%), thrombocytopenia (114,000 platelets/μl), leukocytosis (86.8% neutrophils and 11.5% lymphocytes), and elevated levels of amylase (147 U/L), total bilirubin (6.26 mg/dl), transaminases (aspartate transaminase [AST]: 56 U/L and alanine transaminase [ALT]: 40 U/L), and lactate dehydrogenase (484 U/L). Her glycemia was in normal limits (78 mg/dl). Electrocardiogram showed a slightly sinusal tachycardia and T-wave inversion. Adapted diet, bowel rest, antibiotherapy, and intravenous hydration were administrated, but the patient died 4 days after admission.

**Figure 1:**
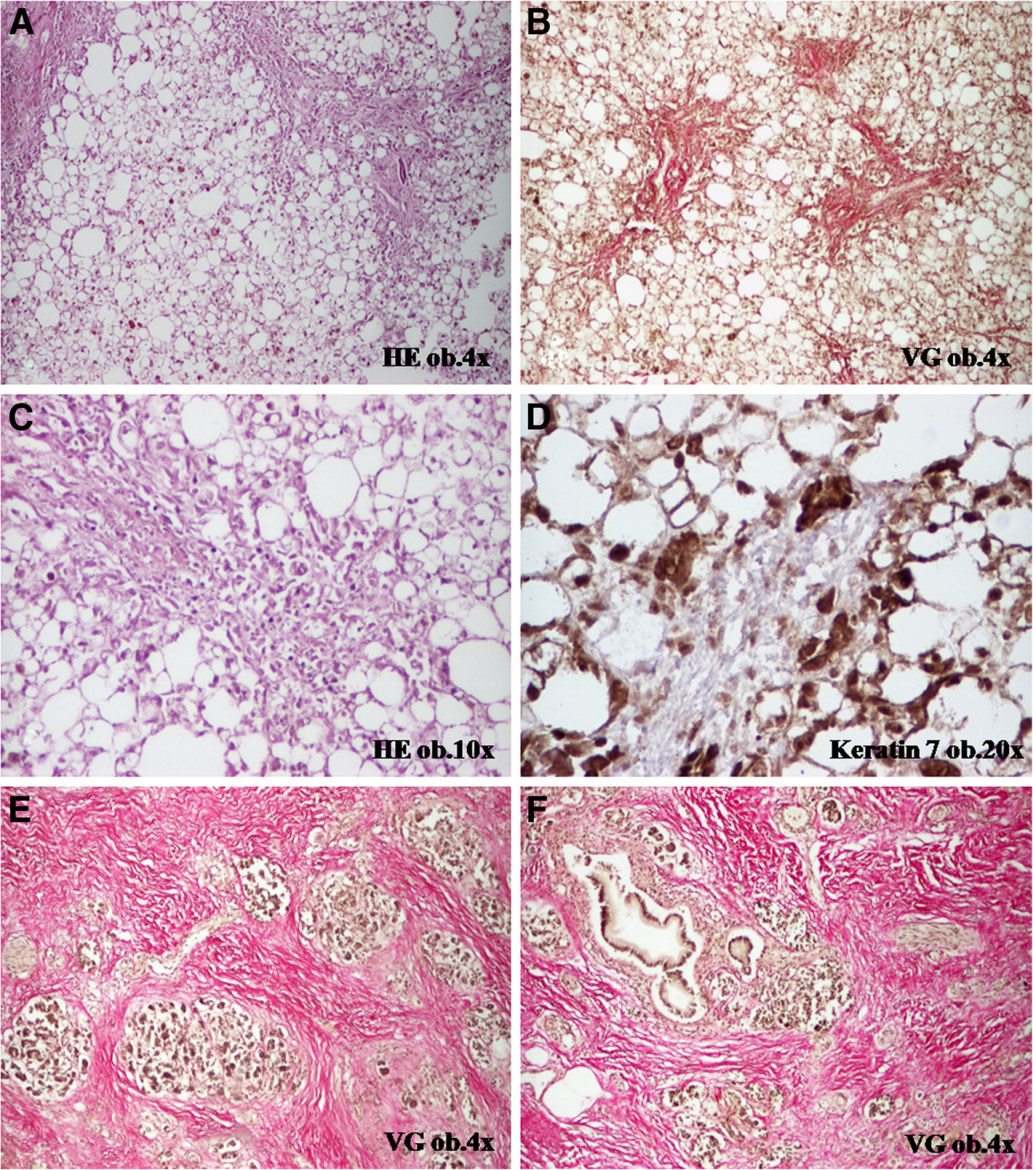
CAPOX-associated toxicity is characterized by steatohepatitis (A-D) and chronic fibrous pancreatitis (E-F).

**Figure 2:**
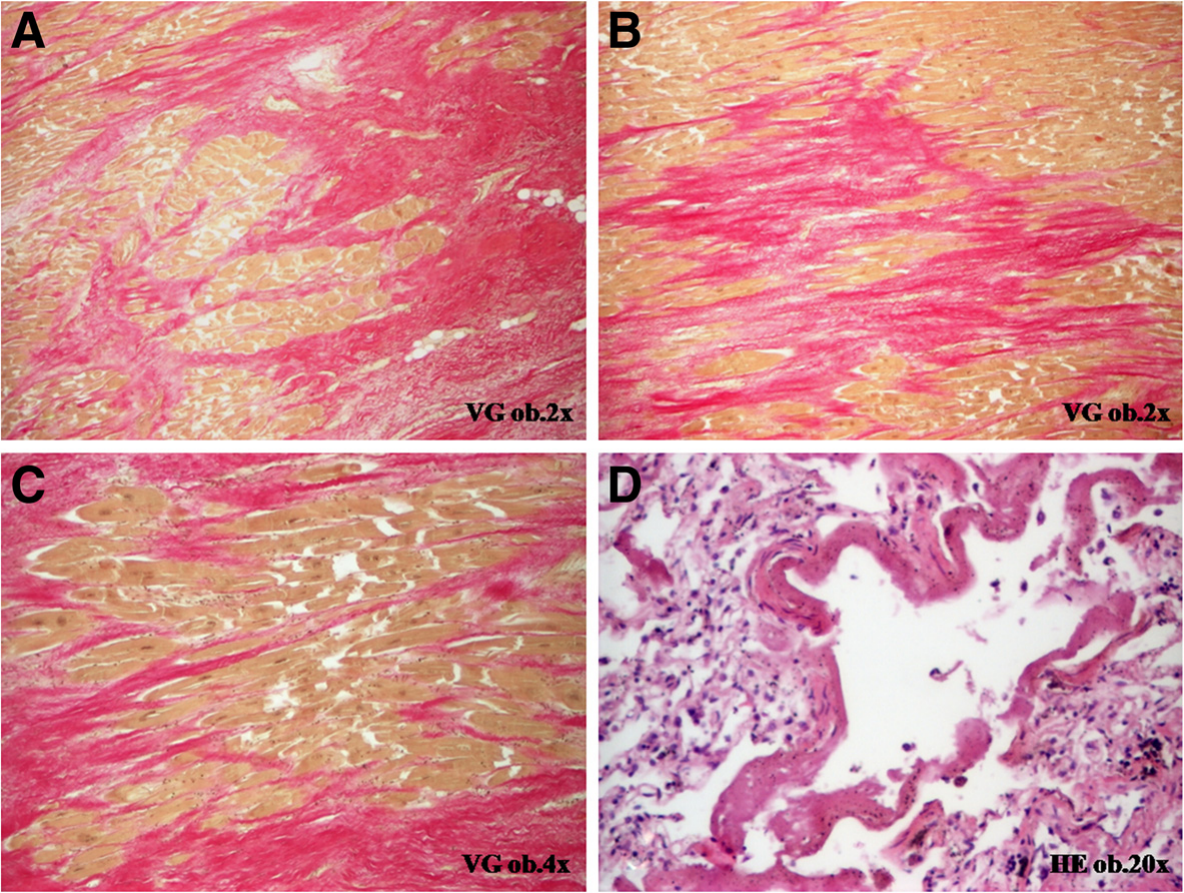
CAPOX regimen is associated with severe myocardial fibrosis (A-C) and pulmonary hyaline membranes (D).

At necropsy, gross and histopathological examination revealed acute non-specific enteritis, chronic pancreatitis with large fibrotic areas and atrophy of the pancreatic parenchyma, and severe steatohepatitis (Figure 1). Most of the hepatocytes were transformed into fatty hepatocytes, the portal spaces were dilated and fibrotic, and the fibrosis was also intralobullary. Within the fibrotic areas, several billiary channels were observed that were marked by keratin 7. The hepatocytes that were connected to the fibrotic septa were also positive at keratin 7 (Figure 1). No vascular changes such as fibrosis of sinusoids and/or veins necrosis of the central hepatocytes were noted. Besides these disorders, fibrinous pericarditis, hydropericardium (300 ml of serous fluid), bilateral hydrothorax (200 ml of serous fluid on both the parts), ascites (400 ml of serous fluid), scleral jaundice, lung dystelectasis with hyaline membranes (Acute Respiratory Distress Syndrome) (Figure 2), and shock kidney were also observed. No tumor recurrences, either node-positive or distant metastases, were evidenced.

A particular histopathological aspect was also observed in the myocardium. Severe diffuse myocardial fibrosis was present in both right and left ventricles, in subendocardial and subpericardial areas (Figure 2). No coronarosclerosis or myocardium hypertrophy was evidenced. The heart weighted 365 g. Her cardiac function was normal before chemotherapy.

Based on the macro- and microscopic features, it was decided that the death was caused by multiorgan failure syndrome, with the main lesion being the chemotherapy-induced cardiotoxicity. The patient’s age, absence of coronarosclerosis or myocardium hypertrophy, and the associated-hepatic and pancreatic lesions proved that the myocardial disorders had toxic and non-ischemic etiology.

## Discussion

Adenocarcinomas of the ampulla of Vater are very rare malignant tumors of the GI tract. They are usually diagnosed in locally advanced or metastatic stages. Being rare tumors, the optimal first-line therapy has not yet been elucidated. One of the commonest first-line chemotherapeutics association is capecitabine in combination with oxaliplatin (CAPOX or XELOX) [4].

Capecitabine is a pro-drug of the cytotoxic agent 5-FU which can be orally administrated and can release FU directly at the tumor site due to increased activity of thymidine phosphorylase in tumor cells, when compared with normal healthy ones, and most of the researchers admit that it presents a favorable side-effect profile [5,7]. However, some toxic effects have been reported such as hand-foot syndrome or palmar-plantar erythrodysesthesia (painful fingers and toes), diarrhea, nausea, vomiting, and mucositis [4,5,8]. In about 60 studies reported to date, cardiotoxicity was infrequently observed, occurring in 1-18% of the cases, being displayed as angina (70%), arrhythmia (10%), myocardial infarction (10%), or cardiogenic shock (10%), without remission after dose reduction or additional medical prophylaxis [9,10]. About 11% of the patients with capecitabine-associated cardiotoxicity were reported to have died [10]. To date, about 25 cases with capecitabine-related hypertriglyceridemia have been reported [5,8], and three of them also presented capecitabine-induced acute necrotizing pancreatitis that required interruption of capecitabine therapy [5,7,8,11]. The pathogenesis of pancreatitis is not well elucidated, and some researchers have mentioned that capecitabine may reduce the activity of lipoprotein lipase and hepatic triglyceride lipase [5,8,12]. Pancreatitis can also be caused by surgery or the tumor location, such as in the reported case. However, no signs of pancreatitis were detected in our patient before this episode. It has also been suggested that the atherosclerotic risk could increase in patients treated with capecitabine over a 6-month period; however, adjuvant administration of fenofibrate could normalize the serum level of triglycerides [8]. In the present case, the patient had chronic pancreatitis with large fibrotic areas and severe atrophy of the pancreatic parenchyma, without atherosclerosis, after five chemotherapy cycles of capecitabine-based regimen.

Oxaliplatin is a third-generation platinum-based alkylating agent that inhibits DNA synthesis in cancer cells [3]. Its side effects mainly include sensory peripheral neuropathy, followed by fatigue, stomatitis, nausea, vomiting, diarrhea, pulmonary fibrosis, gastrointestinal and liver toxicity, ototoxicity, and nephrotoxicity [3,4,13]. The main hepatotoxic features are steatohepatitis, steatosis, and vascular changes displayed as fibrosis of sinusoids as well as central veins and necrosis of the central hepatocytes (sinusoidal obstruction syndrome) [13]. Steatohepatitis without vascular changes was evidenced in our case. Hepatotoxicity seems to be particularly present after more than six cycles of systemic chemotherapy, probably due to increased oxidative stress and direct effect of endotoxins; however, it could rather be a postoperative morbid effect and may not have led to the patient’s death [13]. Hepatotoxicity could be prevented through supplementation of AdoMet (S-adenosylmethionine) in patients treated with oxaliplatin-based regimen [14], probably by increasing the activity of DNA methyltransferases [15]. To date, only one study has reported the possible oxaliplatin-related acute pancreatitis in six GI tumors [3].

In case of CAPOX, the eligible patients should have a good performance status. According to Overman et al. (2009), patients with granulocytopenia (<1.500 granulocytes/mm^3^), thrombocytopenia (<100.000 platelets/mm^3^), and severe anemia (hemoglobin<10 mg/dl) are not eligible [4]. Cardiac disease, brain metastases, renal function impairment (creatinine clearance <30 ml/min), and liver failure (total bilirubin>1.5 mg/dl, albumin>2.5 mg/dl, and elevated levels of transaminases) are also exclusion criteria [4], although some researchers have proved that the combination of oxaliplatin and capecitabine could also be tolerated in patients with hyperbilirubinemia and hepatic dysfunctions, respectively [16].

In one of the largest prospective phase II clinical studies performed with CAPOX in cases of adenocarcinomas of the small bowel and ampulla of Vater, the following related toxicities were observed: fatigue, peripheral neuropathy, nausea, diarrhea, hand-foot syndrome, and hematologic disorders such as neutropenia, anemia, and thrombocytopenia. The researchers proved that CAPOX regimen was well tolerated and highly effective, with an overall response rate of 50% and no treatment-related deaths [4]. Other rare lesions, such as cerebral infarction, stomatitis, hypomagnesemia, hyperbilirubinemia, hyperglycemia, and hypocalcemia were also reported to be associated with combination regimen of capecitabine and oxaliplatin [8,16]. To our knowledge, no cases with CAPOX-induced chronic pancreatitis have been reported till date, and in only three cases, ileitis occurred in patients with metastatic colorectal carcinomas treated with oxaliplatin, capecitabine, and bevacizumab [17].

It must be noted that in the case presented in this study, the CAPOX regimen was highly effective, without relapses and/or metastases; however, fatal signs of toxicity occurred 1 month after completion of the five cycles. Even in young patients with a good performance status, chemotherapy-induced cardiotoxicity, GI, and liver toxicity, as well as pancreatitis should be taken in account.

## Conclusions

Although chemotherapeutics toxicity is a well-known adverse drug reaction, any toxicity should be carefully approached and widely disseminated, to increase general knowledge. The particularity of this case was the multiorgan toxicity that occurred in a young female with a pT4N0-staged carcinoma of the ampulla of Vater treated with questionable maximum dose of XELOX regimen. These fatal toxic associations highlight the necessity of establishing new standardized predictive criteria for postoperative oncological management of non-metastatic locally advanced cancers of the intestine and colon segments, according to gender and functional status, which will be applicable in clinical practice.

## Consent

Written informed consent was obtained from the patient’s relatives for publication of this Case Report and any accompanying images. A copy of the written consent is available for review by the Editor-in-Chief of this journal.

## Abbreviations

ALT: Alanine transaminase; AST: Aspartate transaminase; CAPOX or XELOX: Therapetic regimen that associates oral administration of capecitabine and oxaliplatin; EGFR: Epidermal growth factor; FDA: Food and Drug Administration; FOLFIRI: Therapetic regimen that associates 5-fluorouracil (5-FU) leucovorin [folinic acid], and irinotecan; FOLFOX: Therapetic regimen that associates 5-FU, leucovorin, and oxaliplatin; GEMOX: Therapetic regimen that associates gemcitabine and oxaliplatin; GI: Gastrointestinal.

## Competing interests

The authors declare that they have no competing interests.

## Authors’ contributions

SG wrote the manuscript and carried out the study design. IJ coordinated the study design and the draft the manuscript and carried out the interpretation of the data from literature. MC carried out the histological examination and immunoassays. ZK carried out the oncological management and interpretation of oncological data. LA participated in the interpretation of clinical data. CM carried out the surgical intervention and follow-up and supervised the study design. All authors read and approved the final manuscript.
